# Supplementary material for: Host-virus chimeric events in SARS-CoV2 infected cells are infrequent and artifactual
Source: bioRxiv. 2021 Feb 17:2021.02.17.431704. Preprint. [Version 1] doi: 10.1101/2021.02.17.431704 (PMC7899447; doi:10.1101/2021.02.17.431704)

bioRxiv preprint doi: https://doi.org/10.1101/2021.02.17.431704; this version posted February 19, 2021. The copyright holder for this preprint (which was not certified by peer review) is the author/funder. This article is a US Government work. It is not subject to copyright under 17 USC 105 and is also made available for use under a CC0 license.

Figure legends

Fig. S1. The presence of viral reads and HVC events across infected samples. (**A**) Viral reads in the indicated virus-infected cells as a proportion of the total reads mapped to the chimeric genome. (**B**) HVC reads in the indicated virus-infected cells as a proportion of the total reads mapped to viral genome. (**C**) Dot plots showing the expression of all human genes in SARS-CoV2 infected A549-ACE2 cells ordered by gene expression. Genes with or without HVC events are highlighted with red and blue, respectively.

Fig. S2. Reproducibility of HVC events in SARS-CoV2 infected A549 and A549-ACE2 cells. (**A**) Venn diagrams comparing known splicing, novel splicing and HVC events in SARS-CoV2 infected A549 (top panel) and A549-ACE2 (bottom panel) cells across independent studies. See **Table S1** for the list of independent studies used here. (**B**) Histograms showing the number of reads spanning the junctions of the indicated events.

bioRxiv preprint doi: https://doi.org/10.1101/2021.02.17.431704; this version posted February 19, 2021. The copyright holder for this preprint (which was not certified by peer review) is the author/funder. This article is a US Government work. It is not subject to copyright under 17 USC 105 and is also made available for use under a CC0 license.

Fig. S3. qPCR validation of proposed viral enrichment method. (**A**) Expression of N-protein in control and virus enriched (1 or 2) samples using N1 and N2 qPCR probes recommended by the CDC.

Tables and Table legends

Table S1. SARS-CoV2 infected samples from independent studies used here. See Table S2 for the complete list of individual samples.

Samples	Accession numbers	Attributions
A549-ACE2	GSE147507	Blanco-Melo *et al.*, *Cell* 2020
	GSE154613	Desai *et al.*, *Nat Com* 2020
A549	GSE159191	Weingaren-Gabbay *et al. bioRxiv* 2020
	GSE147507	Blanco-Melo *et al.*, *Cell* 2020
Calu-3	GSE147507	Blanco-Melo *et al.*, *Cell* 2020
	PRJNA665581	Banerjee *et al.*, *Cell* 2020
	GSE148729	Wyler *et al., bioRxiv* 2020
COVID-19 Patients	GSE147507	Blanco-Melo *et al.*, *Cell* 2020
	GSE151803	Schwartz *et al.*
	GSE150316	Desai *et al.*, *Nat Com* 2020

Table S2. The detailed information of all RNA-seq libraries used in this study. (A-B) The detailed information of RNA-seq libraries from SARS-CoV2 infected (A) or other virally infected cells (B) used in this study. The library size, the total number of reads mapped to the human-virus chimeric genome genome, viral genome or HVC reads are reported.

Table S3. Primers/oligoes used in this study. (A) Primers used for qPCR validation. (**B-C**) Oligos used in viral fragment enrichment method 1 (B) and 2 (C), respectively.

bioRxiv preprint doi: https://doi.org/10.1101/2021.02.17.431704; this version posted February 19, 2021. The copyright holder for this preprint (which was not certified by peer review) is the author/funder. This article is a US Government work. It is not subject to copyright under 17 USC 105 and is also made available for use under a CC0 license.

Figure S1

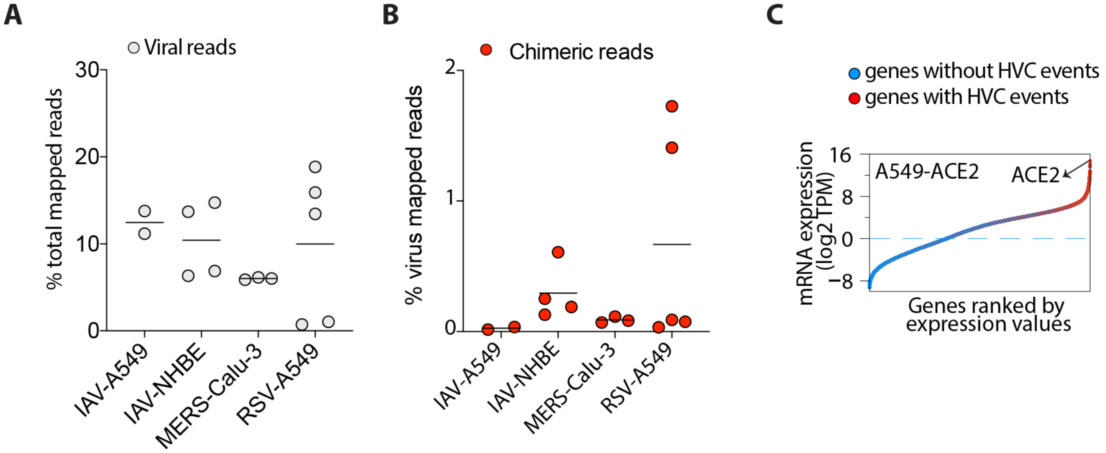

bioRxiv preprint doi: https://doi.org/10.1101/2021.02.17.431704; this version posted February 19, 2021. The copyright holder for this preprint (which was not certified by peer review) is the author/funder. This article is a US Government work. It is not subject to copyright under 17 USC 105 and is also made available for use under a CC0 license.

Figure S2

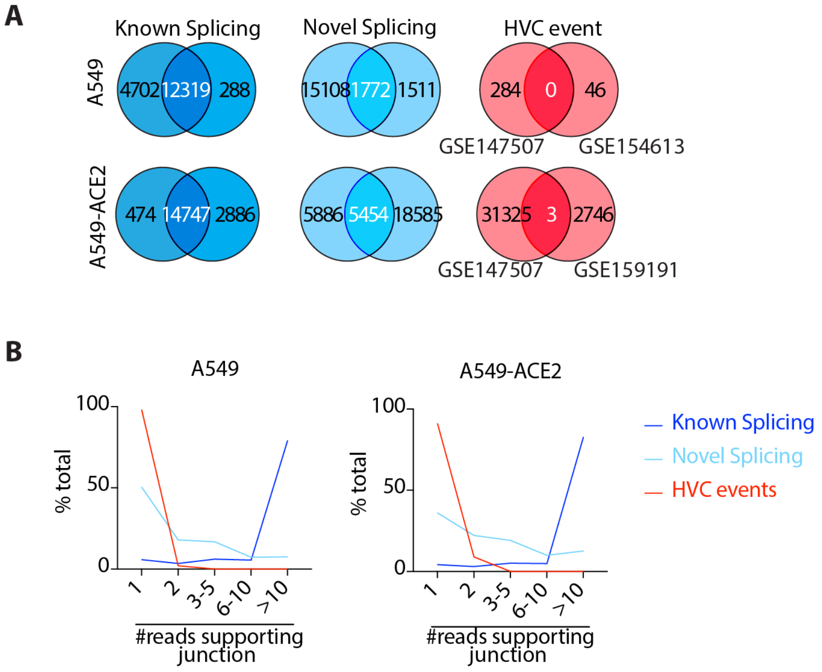

bioRxiv preprint doi: https://doi.org/10.1101/2021.02.17.431704; this version posted February 19, 2021. The copyright holder for this preprint (which was not certified by peer review) is the author/funder. This article is a US Government work. It is not subject to copyright under 17 USC 105 and is also made available for use under a CC0 license.

Figure S3

A

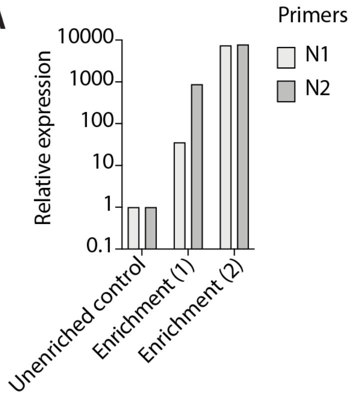

Supplement: 1 [file NIHPP2021.02.17.431704-supplement-1.pdf]
